# Experimental and Numerical Investigation on C/SiC Composite Z-Pinned/Bonded Hybrid Single-Lap Joints

**DOI:** 10.3390/ma14051130

**Published:** 2021-02-27

**Authors:** Yao Wang, Xiaodong Wang, Zhidong Guan, Jifeng Xu, Xia Guo

**Affiliations:** 1 School of Aeronautic Science and Engineering, Beihang University, Beijing 100191, China; wangyaoaria@163.com; 2 Beijing Key Laboratory of Civil Aircraft Structures and Composite Materials, BATRI, Beijing 102211, China; xujifeng@comac.cc; 3 Beijing Center for Physical & Chemical Analysis, Beijing 100094, China; guoxia@bcpca.ac.cn

**Keywords:** 2D C/SiC composites, z-pinned/bonded hybrid joints, progressive damage analysis, numerical model

## Abstract

Z-pinned/bonded joints are great potential connection components that have been used in the 2D C/SiC composite structures; however, the hybrid joints present complex failure mechanism considering the secondary deposited SiC matrix in the clearance. Therefore, the mechanical performance and failure mechanism of the joints are investigated through experimental and numerical methods in this paper. Experiment results show that two peaks exist in the load–displacement curves. The first load peak is 2891–4172 N with the corresponding displacement of 0.10–0.15 mm, and the second load peak is 2670–2919 N with the corresponding displacement of 0.21–0.25 mm. Besides that, the secondary deposited SiC matrix exhibits discrete distribution, and it has significant effects on the failure mechanism. Validated by experimental data, the proposed three-dimensional numerical model based on modified Hashin’s criterion and fastener element can predict the mechanical performance and failure process. The numerical results indicate that the first load peak is dominated by the deposited SiC matrix near the edge, while the second peak is dominated by the z-pin and the SiC matrix near the z-pin. Moreover, the effects of the deposited SiC matrix’s strength and distribution are discussed, which is meaningful to the optimal design of C/SiC composite z-pinned/bonded hybrid single-lap joints.

## 1. Introduction

Carbon fiber reinforced silicon carbide (C/SiC) composite is proven to maintain high specific strength at elevated temperatures, and it has thermal shock resistance with low thermal conductivity and specific heat. Therefore, it has great potential in the application of high-temperature parts of the advanced aircraft structure [1,2,3,4]. Due to the difficulties of productizing large or complex-shaped C/SiC components, the connection technology is significant for the wide application of C/SiC materials. However, it still needs further researches [5].

In recent researches, there has been the online bonded technology via the chemical vapor infiltration (CVI) process to produce structural connection components. This type of connector undergoes secondary infiltration of the matrix after the connector is installed, which not only makes the connector integral, but also enhances the performance of the connection component. The secondary deposition of the SiC matrix causes the joint to have both mechanical connection and adhesive connection characteristics, and the failure situation is thus more complicated.

There have been some researches on the connection component using this online bonded technology. As for the experimental investigation, Zhang has made a systematic contribution to z-pin’s shear failure mechanism and the effects of z-pin’s density and diameter on the mechanical properties of C/SiC single-lap joint [6,7,8]. Zhao focused on the three-bolt joints and proposed the failure pattern of net section tensile fracture at the outermost hole [9]. Among those researches, only a few researches investigated the effect of deposited SiC matrix. The researcher Zongbei He adopted the equivalent bonding efficiency coefficient to characterize the effect of the deposited SiC matrix layer on the bending stiffness of the C/SiC z-pinned two-layer beam [10]. Mao proposed that the behavior of the SiC layer is similar to that of the adhesive layer when studying single-lap C/SiC z-pinned/bonded hybrid joints [11]. In terms of finite element simulation, there are not many models considering the secondarily deposited SiC matrix. Mao [11] used cohesive surface contact to simulate the deposited SiC layer based on a shell-fastener model and discussed the effect of SiC deposition area on the mechanical properties of the joint. However, in our study, the SiC deposition was observed to be distributed in discrete spots, which is not suitable for simulation by the cohesive zone method. Therefore, it is important to characterize the discretely distributed SiC deposition and z-pin failure when studying the joint’s mechanical properties.

In this paper, we carried out a tensile test of a two-dimensional plain-woven C/SiC composite z-pinned/bonded hybrid single-lap joint. Through the analysis of failure morphologies, it was observed that the discrete SiC deposition has an important effect on the failure behavior. Numerical models are established to investigate the failure process of the joint. Then numerical models validated by experiment data can be applied in the discussions about the deposited SiC matrix’s strength and distribution to support the optimal design of the C/SiC z-pinned/bonded hybrid single-lap joint.

## 2. Experiment

### 2.1. Experimental Setup and Procedures

The 2D C/SiC composite used in this research was manufactured by isothermal chemical vapor infiltration (I-CVI). The plain-woven Toray T300-1K 11 × 11 carbon fiber was used as the reinforcement of SiC matrix, and the fiber volume fraction was 47%. For clarity, material orientation 1 was assigned according to the warp fiber bundle.

**Figure 1:**
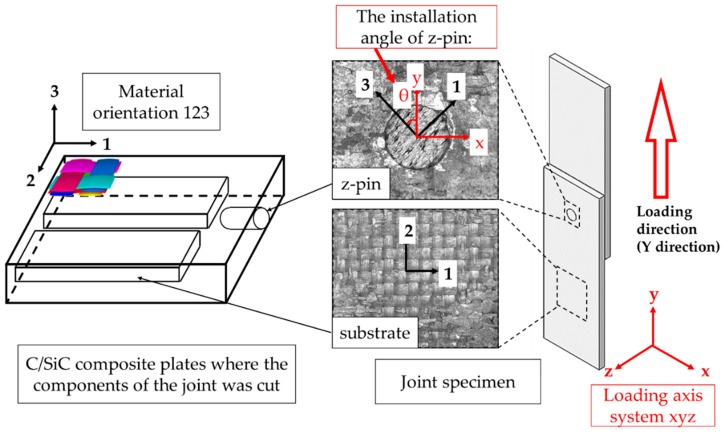
Material directions of C/SiC composite z-pinned/bonded hybrid single-lap joints.

The C/SiC single lap joints were prepared as follows. Firstly, the substrate plates and z-pins were cut and machines from the C/SiC composite plates and the material orientation is shown in Figure 1. Secondly, two substrate plates and a z-pin were assembled shown in. It should be noted that the local material orientation of the substrate plates and the z-pin is different. Here, we defined the assembly angle of z-pin, which is the angle between the loading direction, also the direction Y in the global coordination, and the material orientation 3. As presented in Figure 1, the material coordinate system is illustrated by “123” and marked in black. The global coordinate system is illustrated by “xyz” and marked in red. The assembly angle of each specimen was measured. Finally, the SiC matrix was deposited into the gap of the joints using chemical vapor infiltration (CVI) until the desired densification.

**Figure 2:**
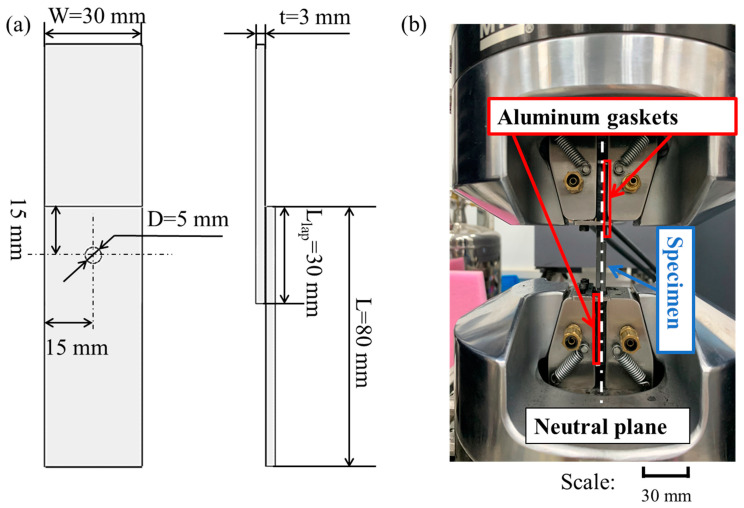
(**a**) Geometric parameters of the tested C/SiC joint; (**b**) Experiment setup.

Four specimens of single-lap z-pinned/bonded hybrid joints, numbered from S1 to S4, were tested in the current work which were provided by Northwestern Polytechnical University. The geometric parameters of the tested specimen are illustrated in Figure 2a. The two substrate plates were 30 mm in width, 80 mm in length, and 3 mm in thickness. The overlap length was 30 mm. And the z-pin was 5 mm in diameter, located in the center of the overlap region.

The tensile experiments of the C/SiC joints were carried out using the MTS (Materials Test Systems) Criterion C45 hydraulic testing machine (MTS Company, Eden Prairie, MN, USA) with a maximum load of 100 kN. To avoid deflection during the test, two aluminum gaskets were applied to ensure that the tensile loading axis passes through the neutral surface of the joint, as shown in Figure 2b. The test was loaded at room temperature under a displacement control model at the speed of 0.5 mm/min until the final failure. The machine displacement and applied load were recorded during the test. After the test, failure morphologies were analyzed by camera photograph (Sony A6400, Tokyo, Japan) and scanning electron microscope (SEM, Quanta 250 FEG, Thermo Fisher Scientific, Hillsboro, OR, USA) technology.

### 2.2. Global Mechanical Response and Strength Analysis

**Figure 3:**
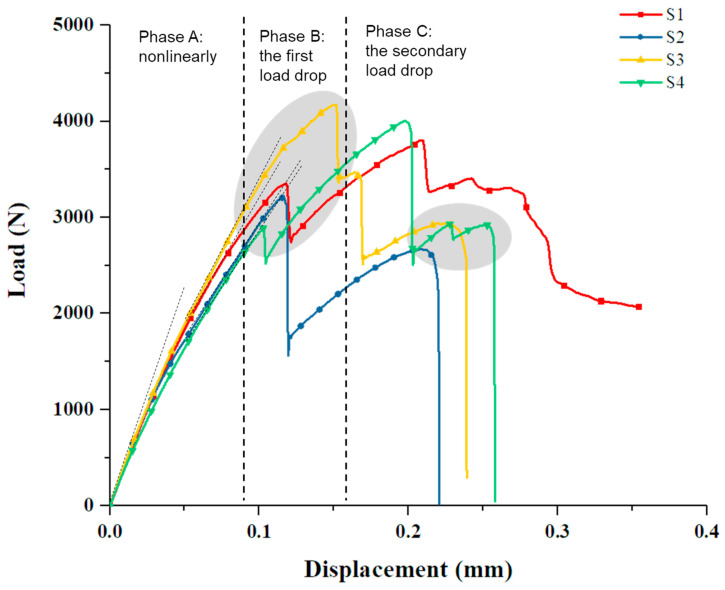
Load and displacement relationship of the test specimens.

The load–displacement relationship is shown in Figure 3, which could be divided into three stages. In phase A, the load increased nonlinearly with displacement. In phase B, the first peak load appeared, with the range of 2891–4172 N, and the corresponding displacement was 0.10–0.15 mm. In phase C, the second or more subsequent load peaks occurred. The load of the specimen S1 decreased gradually, while the specimens S2, S3, and S4 showed a sudden load drop and complete failure. The failure displacement was 0.21–0.25 mm and the corresponding load was 2670–2919 N. The average maximum load of the four test pieces was 3796.35 N. In this experiment, there were two or more load peaks. Similarly, the characteristics of two load peaks also were reported in the bolted/bonded hybrid joint of the epoxy composite [12,13,14].

In addition, the specimen S1 did not show a sudden drop in load at phase C, and the position of its second load peak was not within the failure load–displacement range of the other three specimens. Considering that the z-pin of specimen S1 was bent instead of rupture, it could be inferred that the second load peak was related to the shear failure of the z-pin. The failure mechanism will be discussed in detail in the later section.

The strength of the joint was calculated according to the z-pin’s shear strength.

(1) $$S_{j}=\frac{P_{m}}{A_{j}}=\frac{4P_{m}}{\pi D^{2}}$$

where $P_{m}$ is the maximum load of the joint, and D is the diameter of the z-pin.

The joint shear strength calculated from the maximum load is listed in Table 1, and the average value of the joint strength under tensile loading was 193.35 MPa. Research data showed that the double shear strength of z-pins was 85.8–134.8 MPa (porosity decreases from 23.2% to 14.5%, which also corresponds to density increasing from 1.88 g/cm^3^ to 2.17 g/cm^3^) [15]. It could be concluded that the shear strength of the joint was greater than that of the z-pin, and similar phenomena were reported in the other paper [16]. We believed that not only the z-pin, but also the SiC matrix deposited after assembling could bear the shear load in the C/SiC joint.

### 2.3. Failure Morphologies Analysis

**Figure 4:**
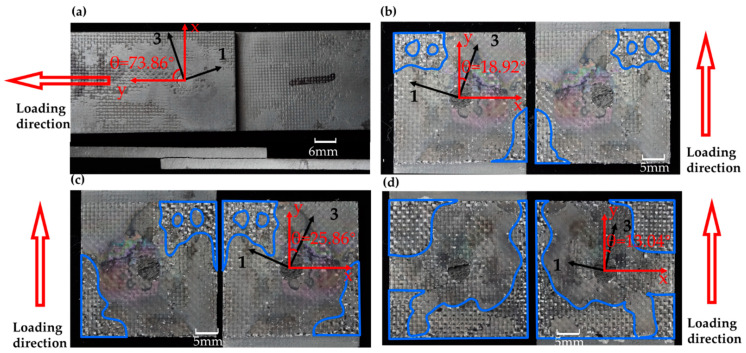
Failure patterns of the four specimens: (**a**) Specimen 1; (**b**) Specimen 2; (**c**) Specimen 3; (**d**) Specimen 4.

The failure morphologies of all the specimens are displayed in Figure 4. For the specimen with a z-pin installation angle near 90°, that was S1, delamination occurred in the overlap region, but the two substrate plates were not completely disconnected, and the z-pin was bent instead of displaying complete rupture. For the specimens with a z-pin installation angle near 0° (i.e., S2, S3, S4), the two substrate plates were separated along the overlap area and the z-pin was ruptured along the delamination surface. On the failure surface, the deposited SiC matrix exhibited nonhomogeneous distribution in the region marked in blue in Figure 4. The secondary SiC deposition was concentrated in the diagonal position or around the overlap area. Both the z-pin and the secondary SiC deposition area were further investigated through SEM technology.

**Figure 5:**
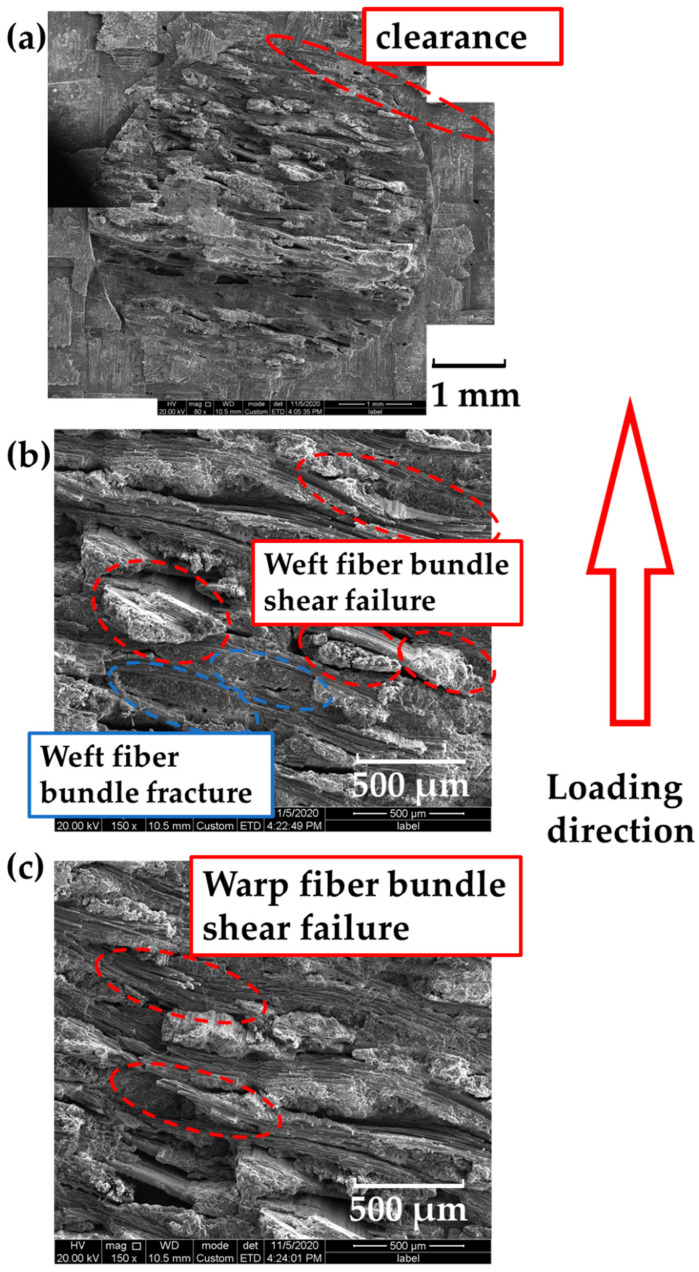
SEM photos of the z-pin on the fracture surface: (**a**) z-pin; (**b**) weft fibers; (**c**) warp fibers.

The microstructure of the z-pin failure is shown in Figure 5. There is a clearance between the z-pin and the hole. Inside the z-pin, some weft fiber bundles were neatly fractured, indicating the characteristics of tensile failure. The fracture surfaces of some weft fiber bundles were at a certain angle to the fiber longitudinal axis, which could be attributed to shear failure. Besides that, the warp fiber bundles mainly exhibited shear failure. To view the z-pin as a whole, the fracture was not in one surface, which could be explained by the stress concentration caused by the woven structure of z-pin.

**Figure 6:**
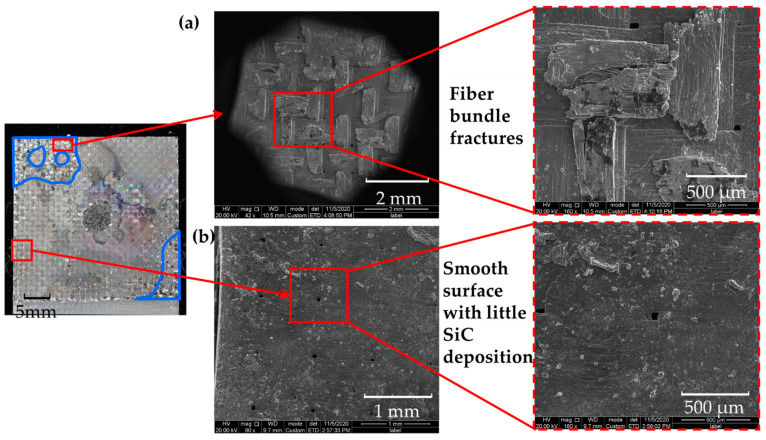
SEM photos of the fracture surface: (**a**) SiC deposition zone; (**b**) smooth surface with little SiC deposition.

From the SEM photograph of the fracture surface of the substrate plate, there was no complete secondary SiC deposition layer formed, but there existed several SiC deposition points distributed discretely, similar to the welding spots. The length of a single deposition point was close to half the wavelength of the woven structure. The width was relatively random, ranging from 1/4 fiber bundle width to nearly the entire fiber bundle width. Fiber fragments could be found on the SiC deposition spot, as shown in the marked area in Figure 6a. In contrast, outside the deposition areas, the fracture surface was relatively smooth with little fiber bundles, as shown in the marked area in Figure 6b. It means that there might be little SiC deposition there or that the bond between the SiC matrix and the fiber was weak.

## 3. Numerical Modelling

### 3.1. Failure Criterion and Material Degradation Models Subsection

The failure criteria of woven ceramic matrix composites are still under research. Current studies on the failure of woven/braided CMC (Ceramic Matrix Composite) materials can be divided into three categories: micro-mechanics theories, multiscale failure theories, and macroscopic failure criterion. For the macroscale model of the joint in this study, the microscale and multiscale failure criteria are too complex to implement. Hashin’s criterion is a failure theory based on physical modes. It can determine not only whether the material is damaged under a given load, but also determine the damage mode, such as fiber tension and compression failure and matrix tension and compression failure. Besides that, Hashin’s criterion has been proven effective to evaluate the damage of the CMC composites [9,17]. Moreover, it is also applied in many researches on the composite joint [18,19,20,21,22]. Here, Hashin-type failure criteria are used in the following numerical models.

Warp fiber bundle tensile/compressive failure:

(2) $$\sigma_{11}\geq 0,\;{\left(\frac{\sigma_{11}}{X_{T}}\right)}^{2}+{\left(\frac{\tau_{12}}{S_{L}}\right)}^{2}+{\left(\frac{\tau_{13}}{S_{T}}\right)}^{2}\geq 1$$

(3) $$\sigma_{11}<0,\;{\left(\frac{\sigma_{11}}{X_{C}}\right)}^{2}\geq 1$$

Weft fiber bundle tensile/compressive failure:

(4) $$\sigma_{22}\geq 0,\;{\left(\frac{\sigma_{22}}{Y_{T}}\right)}^{2}+{\left(\frac{\tau_{12}}{S_{L}}\right)}^{2}+{\left(\frac{\tau_{23}}{S_{T}}\right)}^{2}\geq 1$$

(5) $$\sigma_{22}<0,\;{\left(\frac{\sigma_{22}}{Y_{C}}\right)}^{2}\geq 1$$

Interlaminar tensile/compressive failure:

(6) $$\sigma_{33}\geq 0,\;{\left(\frac{\sigma_{33}}{Z_{T}}\right)}^{2}\geq 1$$

(7) $$\sigma_{33}<0,\;{\left(\frac{\sigma_{33}}{Z_{C}}\right)}^{2}\geq 1$$

The moduli and strength parameters of the C/SiC composite material are listed in Table 2. Here, $X_{T}$ and $X_{C}$ denote the tensile and compressive strength in the material orientation 1, respectively. $Y_{T}$ and $Y_{C}$ denote the tensile and compressive strength in the material orientation 2, respectively. $Z_{T}$ and $Z_{C}$ denote the tensile and compressive strength in the material orientation 3, respectively.$\;S_{12}$ represents in-plane shear strength, and $S_{13}$ and $S_{23}$ are interlaminar shear strength.

Liu [23] proposed a material degradation method based on post-failure behavior in the C/C composite shear model, and it successfully predicted the progressive failure of a cylindrical test specimen. When the tensile failure in direction 1 occurs at a certain element, the element can no longer resist tension along direction 1 and shear in the 1–2 and 1–3 plane. Therefore, ***E*_11_**, ***G*_12_**, and ***G*_13_** are reduced to zero. To solve the convergence problem, these moduli are reduced to 0.01 times of original value here to represent a sudden decrease in stiffness. When compression failure in direction 1 occurs at a certain element, *σ*_11_ of the element is close to the undamaged condition. In this state, the shear stress is formed by the friction force of the fracture surface, which is relevant to shear strain and *σ*_11_. To simplify the simulation, stiffness degradation is employed to model the shear response and the degradation coefficient is recommended to be 0.1 in this model. The material degradation models are given in Table 3.

### 3.2. SiC Deposition Zone Modelling

**Figure 7:**
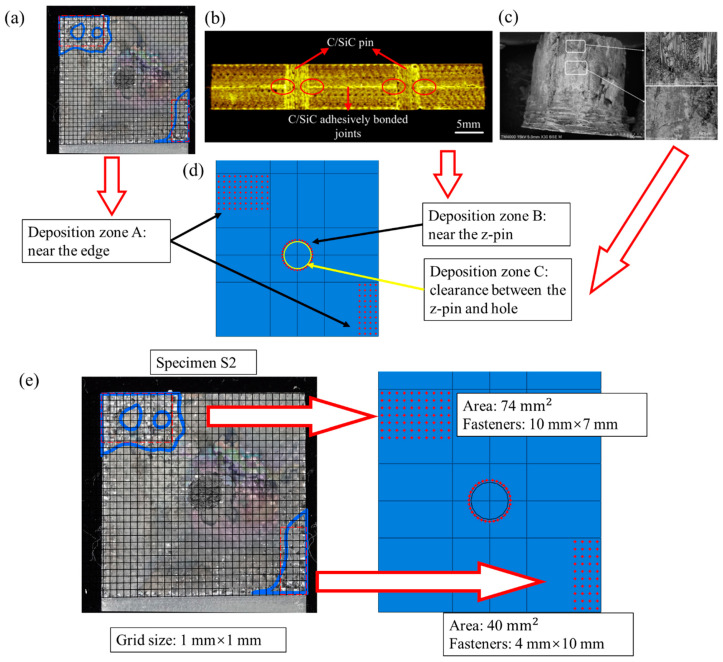
Three types of SiC deposition zones in the C/SiC z-pinned/bonded hybrid single-lap joint: (**a**) deposition zone A: near the edge; (**b**) deposition zone B: near the z-pin [10]; (**c**) deposition zone C: clearance between the z-pin and hole [27]; (**d**) numerical model; (**e**) Fasteners’ distribution with similar area in deposition zone A.

During the secondary deposition after joint assembling, SiC matrix is formed between the two substrate plates. Moreover, as the SiC matrix is introduced by the CVI process, it can be enhanced by the flow path of the reaction gas. The clearance between z-pin and hole can provide a flow path. Hence, SiC deposition is formed in the mentioned clearance and on the substrate near the z-pin. The SiC deposition area of the C/SiC joint can be divided into three parts, as shown in Figure 7, the area on the substrate plate near the edge (deposition zone A, DZA), the area on the substrate plate near the z-pin (deposition zone B, DZB), and the area in the clearance between the z-pin and hole (deposition zone C, DZC). The deposition zone A can be seen from the SEM pictures that the SiC deposition is discretely distributed like welding spots. It is not appropriate to be modeled with the continuous cohesive layer. Enlighted by the numerical modelings of spot welding [24,25,26], fastener elements are applied to simulate discretely distributed SiC deposition spots. The space of the adjacent fastener is 1 mm, obtained from multiple measurements in the SEM picture and rounding the average. Because the actual deposition areas have complicated shapes, the rectangular array of fastener elements with a similar area is used instead. The deposition zone B is represented by a set of closely arranged fastener elements. The deposition zone C can be seen to be composed of relatively continuous SiC and void [27], which is represented by a cohesive layer here. The gap between the two substrate plates is 0.05 mm, and the clearance between the z-pin and hole is 0.05 mm [9].

The attachment points were defined between the two substrate plates. The connection between the attachment points was defined using Point-Based Fasteners with the Connector response of “Bushing”. The Bushing connection can define stiffness and failure. The fasteners are assumed to have a linear stress–strain response, and the stiffness of fasteners is calibrated from specimen S2’s experiment data. For the fastener elements in the DZA area, the secondary bending of the joint may cause two failure modes, interlaminar tensile failure, and shear failure. The interlaminar tensile strength is 18 MPa [28], and the interlaminar shear strength is 74 MPa, the sliding shear strength of the SiC matrix [11]. Once certain stress reaches the corresponding strength, the entire fastener element fails and releases all the stress. For the fastener elements in the DZB area, the secondary bending phenomenon is not obvious, only the shear failure is considered, and the shear strength is fitted by the load–displacement curves of the experiment for lack of data and the value is 110 MPa.

For the cohesive element in the DZC area, the traction–separation constitutive relationship is adopted, and the failure is determined by the quadratic nominal stress criterion. The energy-based damage evolution is adopted. The parameters of cohesive elements, listed in Table 4, are explained and discussed.

Similar to the adhesively bonded joint models proposed previously [29,30], the stiffness of the cohesive elements used to represent the adhesive material layer can be estimated using the Young’s modulus of the SiC matrix and thickness of the SiC deposition. The experimental value of SiC matrix modulus is 113.3 GPa [31]. Due to a large number of pores, according to the experimental load–displacement curve of S2, the stiffness of the cohesive element here is estimated as 0.3 times the SiC matrix modulus.

**Figure 8:**
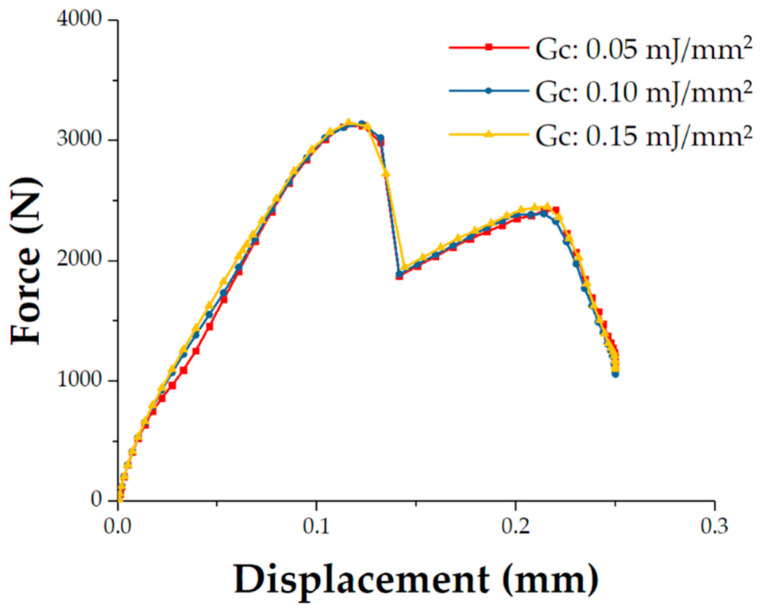
The load–displacement curves of the established numerical model with different critical energy release rate of the cohesive elements.

It is difficult to determine the critical energy release rate for the mechanical properties of the SiC deposition. The critical energy release rate of carbon reinforced SiC matrix composite is reported to be 0.1–0.53 mJ/mm^2^ [32,33,34,35]. Here, as shown in Figure 8, the models with different critical energy release rates (0.05 mJ/mm^2^, 0.1 mJ/mm^2^, and 0.15 mJ/mm^2^) are calculated respectively. The critical energy release rate can affect the initiation stage of the displacement-load curves of tensile joint, but has little effect on the failure load and displacement. It can be inferred that the failure of the joint is insensitive to the critical energy release rate within the range of 0.05–0.15 mJ/mm^2^. The model proposed in the manuscript focuses on the failure strength or displacement of the C/SiC joint, so the critical energy release rate is chosen as 0.1 mJ/mm^2^.

In addition, studies [36,37,38] have shown that the mesh size of cohesive elements can affect the accuracy of the results. The material characteristic length $l_{mat}$ is predicted as [37,38]:

(8) $$l_{mat}=\frac{EG_{c}}{{\left(\tau^{0}\right)}^{2}}$$

where E is the Young modulus of the material, $G_{c}$ is the critical energy release rate, and $\tau^{0}$ is the maximum interfacial strength. Hence, $l_{mat}$ can be obtained, and it is 37.66 mm. The mesh size of the cohesive elements used in the model is 0.2 mm, which can provide accurate results.

Here, viscous regularization is used to solve the convergence problem. The viscosity coefficient adopts 0.001 as recommended in the published paper [7].

### 3.3. Finite Elements and Boundary Conditions

**Figure 9:**
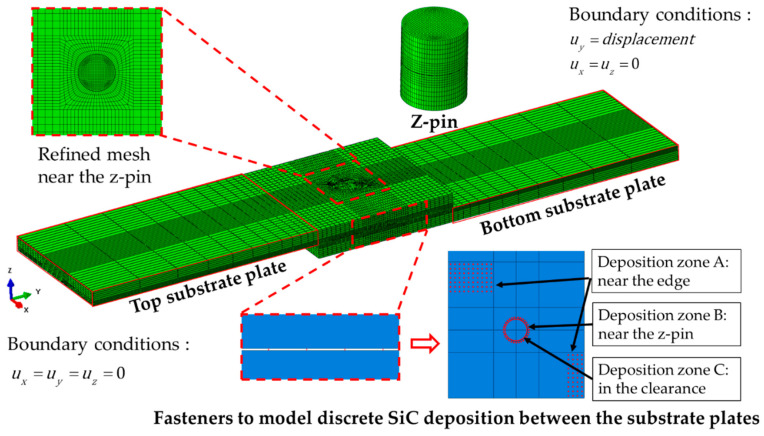
Numerical model description of the C/SiC z-pinned/bonded hybrid single-lap joint.

The C/SiC z-pinned/bonded hybrid joint is composed of two substrate plates, z-pin, and fastener elements to represent SiC deposition and cohesive layer between the z-pin and hole. As shown in Figure 9. The installation angle of the z-pin is 18.13°, identical to the specimen S2. The two substrate plates and z-pin are constructed by linear hexahedral elements (C3D8R). The mesh of the substrate is refined near the z-pin and near the 1/3 thickness of the loading surface. After the mesh independence check, the mesh size of the z-pin is determined to be 0.2 mm.

The failure criteria and material degradation in Section 3.1 are embedded into the software Abaqus/Standard with a user-defined Fortran code USDFLD to simulate the progressive failure behavior of C/SiC z-pin and substrate plates. The boundary conditions are defined with coupling constraints to ensure that the load is on the neutral surface of the joint. Specifically, one end of the joint is fully constrained, the other end of the model is subjected to a displacement of 0.25 mm along the Y direction.

## 4. Failure Mechanism Analysis and Discussion

### 4.1. Numerical Results and Validation

**Figure 10:**
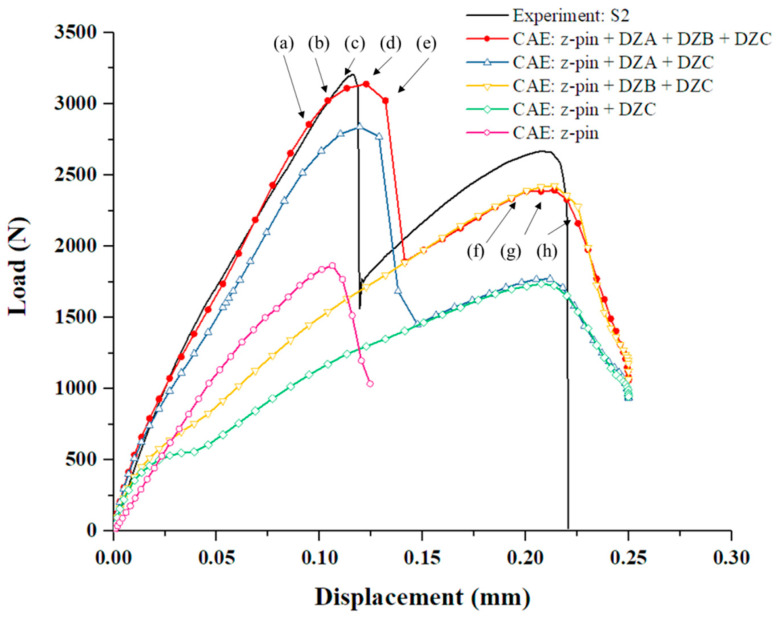
Load–displacement curves of the numerical results with different SiC deposition zones. (Point (a)–(h) represent the key moments of the failure process, and are explained in Section 4.2).

The numerical results of the load–displacement curve are compared with the typical experimental data to validate the proposed finite element model. For the model with z-pin and three typical deposition zones, the load–displacement curve exhibits two load peaks as shown in Figure 10. The first peak load is 3139.65 N, the corresponding displacement is 0.1227 mm, and the error from the experimental data (3205.79 N, 0.1163 mm) is 2.06% and 5.50%, respectively. The second peak load is 2393.75 N, the corresponding displacement is 0.2140 mm, and the error from the experimental data (2669.89 N, 0.2088 mm) is 10.34% and 2.49%, respectively. Therefore, the established finite element model can be proven to validate the C/SiC joint.

Besides that, after proven valid, the numerical model is modified to discuss the influence of the three types of SiC deposition zones on the load–displacement curve as shown in Figure 10. First, the clearance between the z-pin and hole delays the failure displacement of the second load peak, yet has little effect on the value of the second peak load. Secondly, the SiC matrix in DZB near the z-pin will increase the second peak load, but the load–displacement curve presents a single peak. Moreover, considering the SiC matrix in DZA between the two substrate plates, the curve shows a double-peak characteristic. It can be concluded that the first peak of the load–displacement curve corresponds to the failure of the SiC matrix in DZA (the region of substrate plate near the edge), and the second peak corresponds to the failure of the z-pin and the SiC matrix in DZB (the region of substrate plate near the z-pin).

### 4.2. Failure Mechanisms of the C/SiC Z-Pinned/Bonded Hybrid Joint

**Figure 11:**
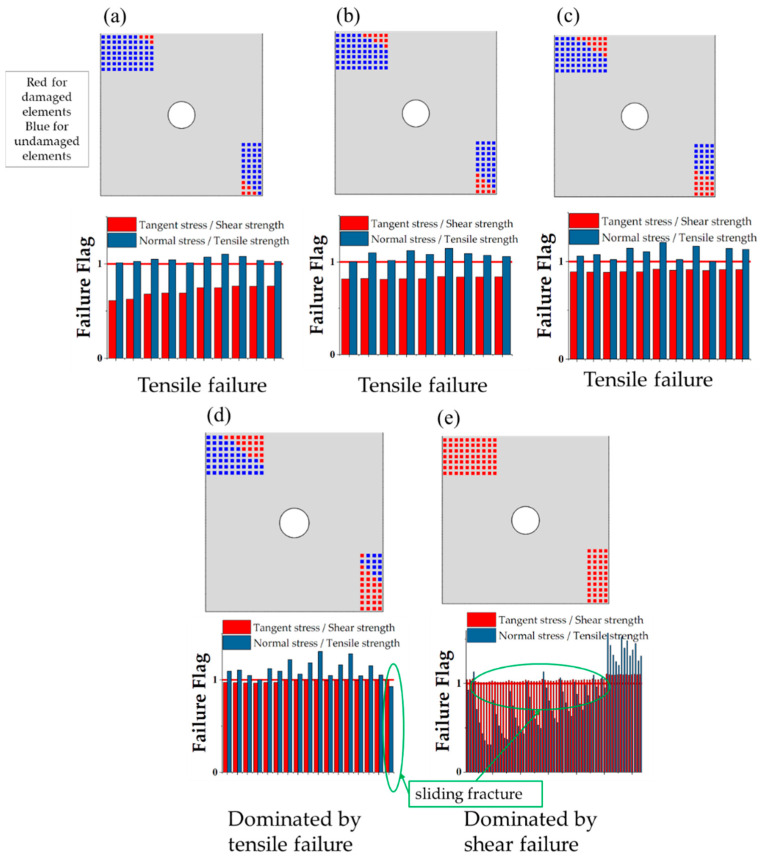
Progressive failure and failure mode of the SiC matrix in deposition zone A (DZA). (Point (**a**)–(**e**) represent the key moments of the failure process as illustrated in Figure 10).

**Figure 12:**
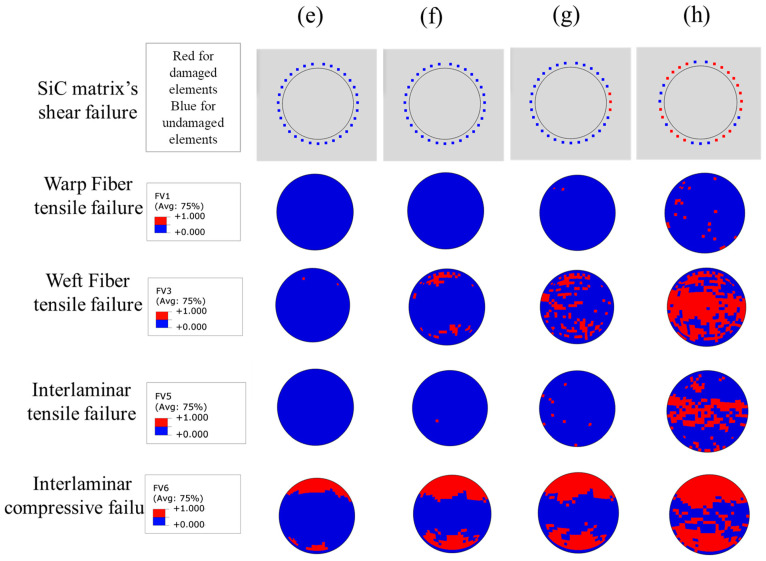
Progressive failure of the SiC matrix in deposition zone B (DZB) and the z-pin. (Point (**e**)–(**h**) represent the key moments of the failure process as illustrated in Figure 10).

Based on the numerical model, the failure development of C/SiC z-pinned/bonded hybrid joint under tensile loading is traced and analyzed in Figure 11 and Figure 12. According to the failure characteristics, some key points are selected near the two load peaks in reference to Figure 10. Firstly, as the load increases, the SiC matrix in DZA begins to fail near the edge of the overlap region at point (a). To determine the failure mode, the stress–strain curve of each fastener element that represents SiC deposition spot is derived. And the tensile failure flags of each SiC deposition spot are calculated as the tensile stress divided by tensile strength and the shear failure flags are processed similarly. The tensile flag is blue while the shear flag is red, as shown in Figure 11. It can be seen that the failure mode at point (a) is tensile failure. This is attributed to the secondary bending of the substrate plates, and the resulting out-of-plane displacement is more severe near the edge of the overlap region along the loading direction. Then, the failure range of the SiC matrix gradually expands toward the center of the overlap region, and the failure mode is dominated by tensile failure, as shown in Figure 11 from point (b) to (d). The stiffness of the joint is decreasing accordingly. Until point (e), the remaining SiC matrix in DZA fails completely and the main failure mode is switched to shear fracture. Since the bearing capacity of SiC deposition zone A is greatly reduced due to the secondary bending effect while the load continues to grow, the shear load of the SiC deposition points is redistributed and rapidly reaches the shear failure strength. It can be observed that the first load peak appeared just before SiC matrix completely fails. At the first load peak, neither SiC matrix in DZB nor the z-pin failed. Therefore, the first load peak is dominated by the SiC matrix in DZA. In addition, the finite element results show that the secondary bending phenomenon causes the SiC matrix in DZA to exhibit tensile failure before the shear failure, thereby limiting the first peak load of the joint.

Figure 12 shows the failure process of the z-pin and SiC matrix in DZB. Here, the middle section of the z-pin is observed, which is also on the loading plane. Although the interlaminar compressive failure of the z-pin can be observed at the first load peak, the weft fibers which mainly transfer the shear load are intact mostly at point (e). To clarify, the shear failure is integrated into tensile failure in Hashin’s criteria. The field variable 1 (FV1) denotes the tensile failure of the warp fiber, and the field variable 3 (FV3) denotes the tensile failure of the weft fiber. The field variable 5 (FV5) and field variable 6 (FV6) are interlaminar tensile failure and compressive failure, respectively. With the applied load increasing to point (f), weft fiber failure of the z-pin appears at the edge along the loading direction, before the failure of SiC matrix in DZB. When the load reaches the second peak at point (g), the failure of the z-pin is dominated by weft fiber fracture, and the interlaminar matrix tensile failure occurs at the center of the z-pin. Thus, the failure of SiC deposited near the z-pin begins at the center area. After the second load peak at point (h), most of the SiC matrix in DZB failed and the middle section of z-pin is full of weft fiber failure. Therefore, the second load peak is dominated by the SiC matrix in DZB and the z-pin.

### 4.3. The Effect of the Deposited SiC Matrix’s Strength on the Joint’s Mechanical Behavior

The SiC matrix of the joint formed by the secondary CVI process exhibits randomness in distribution and material properties. The effect of the deposited SiC matrix’s strength on the C/SiC joint mechanical performance is explored based on the above model. Since the strength of the deposited SiC matrix is affected by the production process parameters, there is no specific relevant data, so a coefficient of variation is adopted here to characterize the changing of SiC matrix’s strength compared to the value used in the model (Section 3.2). The SiC matrix in DZA and DZB are analyzed respectively as follows.

**Figure 13:**
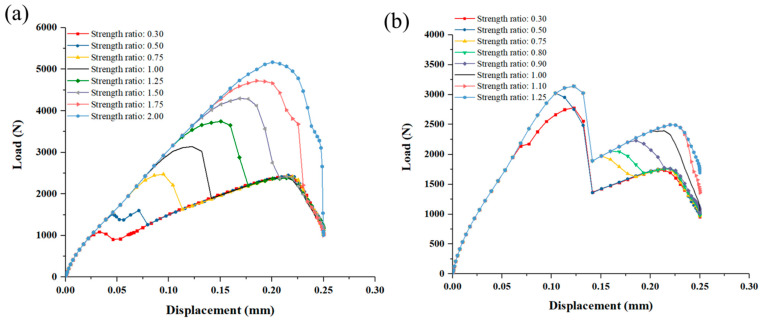
Load and displacement curves of different strength of: (**a**) Deposited SiC matrix in DZA; (**b**) Deposited SiC matrix in DZB.

**Figure 14:**
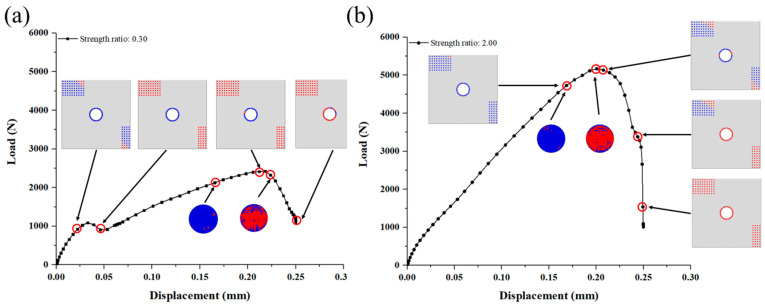
Progressive failure of the joint when the strength of SiC matrix in deposition zone A (DZA) is (**a**) 0.3 times; (**b**) 2.00 times the original value.

As for the SiC matrix in DZA, the value of the first load peak increases as the deposited matrix’s strength increases, shown in Figure 13a. When the SiC matrix’s strength is 0.3 times of the original model (that is 5.4 MPa for the interlaminar tensile strength and 22.2 MPa for the shear strength), there are still two load peaks, and the first load peak is smaller than the second one. The failure process in Figure 14a shows that the first load peak is dominated by the failure of the SiC matrix in DZA, and the second load peak is dominated by the weft fiber fracture of the z-pin. After the second load peak, the SiC in the DZB area gradually fails. At this time, the second load peak is the maximum load for the joint, and the strength of the joint at this time is determined by the strength of the z-pin and the SiC matrix in DZB. When the strength is twice as the original model (that is 36 MPa for the interlaminar tensile strength and 148 MPa for the shear strength), there is only one load peak. The failure process in Figure 14b displays that the z-pin fails at the load peak while most of the SiC matrix in DZA remains intact. The strength of the joint is dominated by the SiC matrix of DZA.

**Figure 15:**
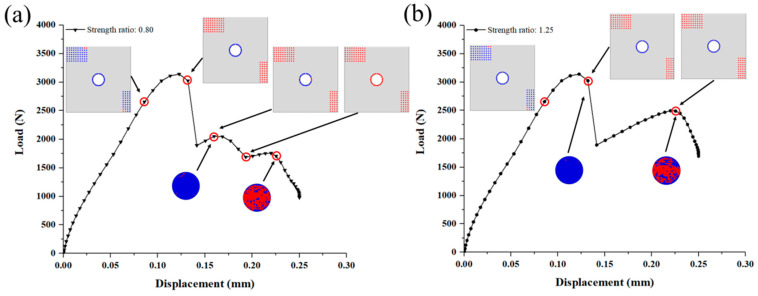
Progressive failure of the joint when the strength of SiC matrix in deposition zone B (DZB) is (**a**) 0.8 times; (**b**) 1.25 times the original value.

As for the SiC matrix in DZB, the effect could be complex as shown in Figure 13b and Figure 15. Firstly, when the SiC matrix’s shear strength in DZB is less than 0.5 times (i.e., less than 55 MPa), the first peak load increases with the increase of matrix strength. When the strength is greater than 55 MPa, however, the first load peak is hardly affected by it. Secondly, when the SiC matrix’s shear strength in DZB is between 0.75–0.9 times, the load–displacement curve displays three peaks. The second load peak increases with the increase of DZB matrix’s strength, while the third load peak is not affected by the strength. This is because the second load peak is dominated by the failure of the SiC matrix of DZB, while the third load peak is dependent on the z-pin’s strength. Lastly, when the SiC matrix’s shear strength in DZB is greater than the original value in Section 3.2, two load peaks appear in the load–displacement curve, and the second load peak increases as the strength increases.

**Figure 16:**
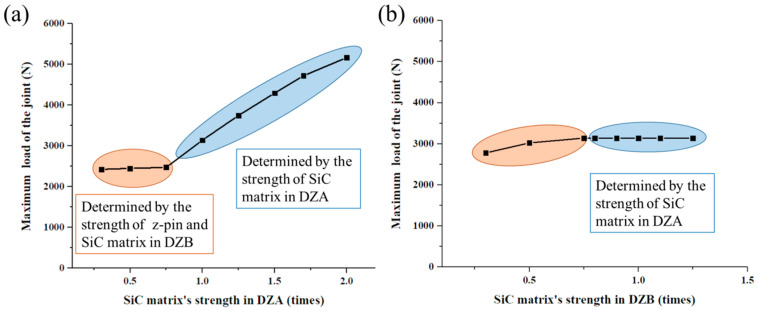
The relationship of the joint’s maximum load and the deposited SiC matrix’s strength in (**a**) DZA; (**b**) DZB.

The relationship of the joint’s maximum load and the deposited SiC matrix’s strength are summarized in Figure 16. The maximum load of the joint can be affected obviously by the SiC matrix’s strength in DZA rather than DZB. When the SiC matrix’s strength in DZA is less than 0.75 times the original value (13.5 MPa for interlaminar tensile strength and 55.5 MPa for interlaminar shear strength), the maximum load of the joint is determined by the strength of the z-pin and SiC matrix in DZB. When the SiC matrix’s strength in DZA is greater than 0.75 times the original value, the maximum load of the joint increases almost linearly as the strength. which makes the deposited SiC matrix a crucial factor determining the mechanical properties of the joint.

### 4.4. The Effect of the Deposited SiC Matrix’s Distribution on the Joint’s Mechanical Behavior

**Figure 17:**
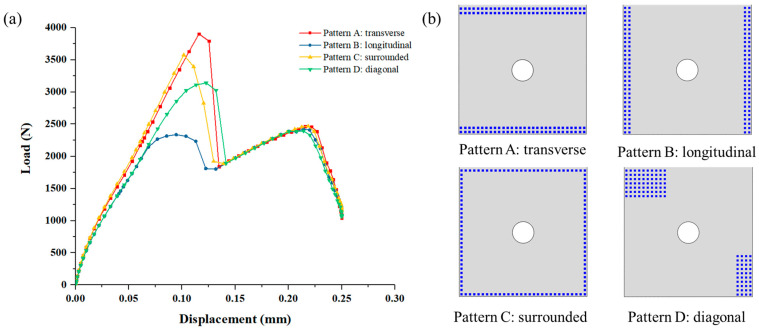
(**a**) Load and displacement curves with different distributions of the deposited SiC matrix in DZA; (**b**) Four distribution patterns of the deposited SiC matrix in DZA.

Numerical models with different deposited SiC matrix patterns in DZA are established, and they are transverse pattern, longitudinal pattern, surrounded pattern, and the diagonal pattern discussed previously. Figure 17 shows the effect of the deposited SiC matrix’s distribution on the joint performance. The model with longitudinal pattern shows the smallest peak load, which is 22.79% lower than the diagonal model, and the maximum load occurs at the second load peak instead of the first. While the transverse model presents the largest peak load, which is 24.27% higher than the diagonal model, and 13.89% higher than the surrounded model. And the maximum load of these three all appear at the first load peak. Moreover, the distribution of the deposited SiC matrix in DZA mode has little effect on the second load peak.

**Figure 18:**
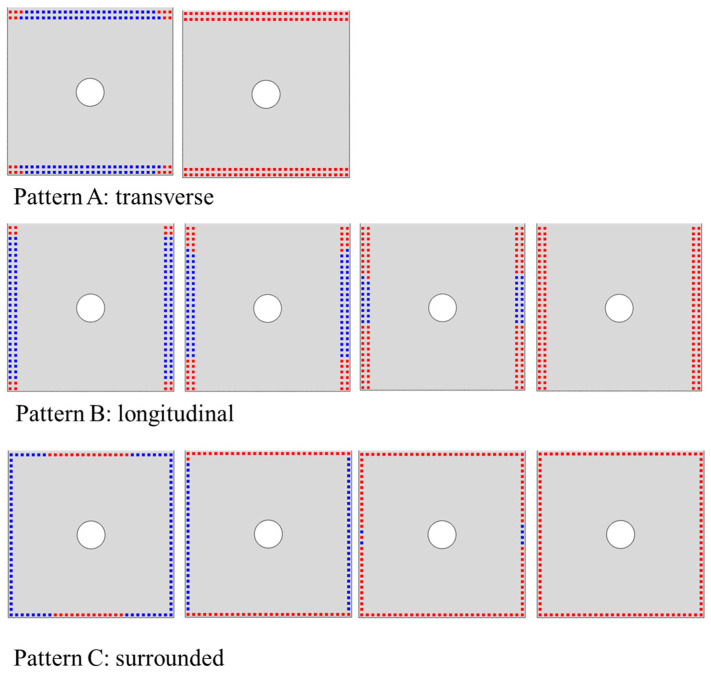
Progressive failure of the deposited SiC matrix in DZA.

As illustrated in Figure 18, in the transverse model, the SiC matrix reaches the tensile strength initially at the four corners of the overlap area, and then the other matrix deposition spots quickly fail due to shear stress. Similarly, in the longitudinal model, the failure also starts the four corners of the overlap area and then gradually expands to the center. For the surrounded model, the failure of SiC matrix initiates at the center of the edge that is perpendicular to the loading. Therefore, the tensile stress caused by secondary bending can severely affect the failure of SiC matrix. The longitudinal model of C/SiC hybrid joint lacks SiC matrix reinforcement where the maximum displacement outside the secondary bending plane is. It can explain the lowest joint strength for the longitudinal distribution while the highest joint strength for the transverse distribution.

Furthermore, the effect of infiltration depth of SiC matrix on the joint’s mechanical performance is discussed based on the surrounded model. Because, ideally, the chemical gas in the CVI process should penetrate between the two substrate plates from all around edges of the overlap area thus generates the SiC matrix. The distance between adjacent deposition points is 1 mm, the same as the model in Section 3.2.

**Figure 19:**
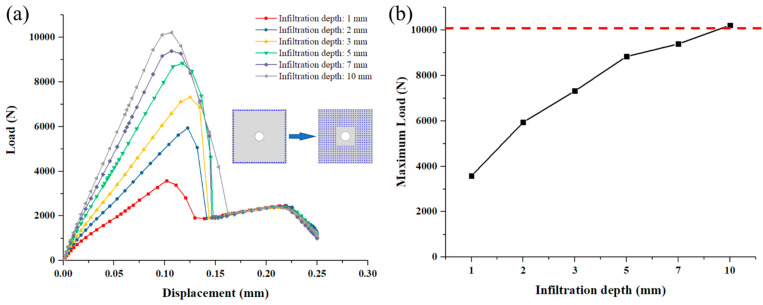
(**a**) Load and displacement curves with different infiltration depths of the deposited SiC matrix in DZA; (**b**) Relationship between the joint’s maximum load and the infiltration depth of the deposited SiC matrix in DZA.

It is illustrated in Figure 19 that, as the infiltration depth of SiC matrix, the first load peak increases significantly while the second load peak hardly changes. When the infiltration depth increases from 1 mm to 2 mm, the maximum load increases from 3575.61 N to 5938.53 N by 66.08%. When the infiltration depth is increased from 5 mm to 10 mm, the maximum load increases from 8835.71 N to 10,208.7 N merely by 15.54%. So, the increase in the infiltration depth can effectively cause the maximum load of the joint to increase by a decreasing extent. Although the complete infiltration of SiC matrix can greatly enhance the strength of the C/SiC joint, it is unrealistic considering the CVI process cost and production time. Researches on relevant manufacture parameters would be desired to explore the optimal infiltration depth.

## 5. Conclusions

To explore the potential of ceramic matrix composites in advanced ultra-temperature aircraft, the mechanical properties and failure mechanism of the connection components deserve further researches. In this study, the C/SiC z-pinned/bonded hybrid joint under tension is studied through experimental and numerical methods. The effect of the SiC matrix deposited from secondary infiltration is also studied.

Firstly, the tensile test of C/SiC z-pinned/bonded hybrid joint was conducted. The load–displacement curves exhibited the two-load-peak characteristic. The first load peak was 2891–4172 N, and the corresponding displacement was 0.10–0.15 mm. The second load peak was 2670–2919 N, and the corresponding displacement was 0.21–0.25 mm. The average strength of the joint was 193.35 MPa. The failure morphologies showed that the z-pin of specimens with an installation angle near 0° was ruptured. On the delamination surface, the deposited SiC matrix exhibits nonhomogeneous distribution mainly in the diagonal region of the overlap area.

Secondly, a three-dimensional model of C/SiC z-pinned/bonded hybrid joint is established, considering the z-pin and three types of deposited SiC matrix. Fastener models are applied to simulate the secondary deposition spots between the two substrate plates. The numerical results are well matched to the experimental data. Besides that, the damage process of the model shows that the first load peak is dominated by the deposited SiC matrix near the edge while the second load peak is dominated by the z-pin and the SiC matrix near the z-pin. The model also proves that secondary bending of the substrate will cause the SiC matrix near the edge to exhibit tensile failure before the shear failure, thereby limiting the first peak load of the joint.

Thirdly, the effect of the deposited SiC matrix’s strength on the C/SiC joint’s mechanical performance is explored based on the above numerical model. The maximum load of the joint can be affected obviously by the strength of the SiC matrix near the edge rather than near the z-pin. When the strength of the SiC matrix near the edge is less than 0.75 times the original value (13.5 MPa for interlaminar tensile strength and 55.5 MPa for interlaminar shear strength), the maximum load of the joint is determined by the strength of z-pin and SiC matrix nearby. When it is greater than 0.75 times the original value, the maximum load of the joint increases almost linearly as the strength, which makes the deposited SiC matrix a crucial factor determining the mechanical properties of the joint.

Finally, four types of deposited SiC matrix patterns between the substrate plates are discussed, they are transverse pattern, longitudinal pattern, surrounded pattern, and diagonal pattern. The transverse pattern model shows the highest joint strength while the longitudinal model shows the lowest, which can be explained by the lack of SiC matrix reinforcement where the maximum displacement outside the secondary bending plane is. Furthermore, the effect of infiltration depth of SiC matrix on the C/SiC joint’s mechanical performance is studied based on the surrounded model. The results exhibit that as the infiltration depth of SiC matrix increases, the first load peak increases significantly while the second load peak hardly changes. However, the increase in the infiltration depth can effectively cause the maximum load of the joint to increase by a decreasing extent. Therefore, there may exist an optimal infiltration depth to not only enhance the strength of C/SiC joint, but also control the manufacturing cost.

## Figures and Tables

**Table 1:** Failure properties of the C/SiC z-pinned/bonded hybrid single-lap joint.

Test Specimens	Displacement at the Maximum Load (mm)	Maximum Load (N)	Shear Strength of Joint $S_{j}$ (MPa)	Failure Pattern	Assembly Angle of the Z-Pin
S1	0.2090	3801.37	193.60	z-pin bent	73.86°
S2	0.1163	3205.79	163.27	z-pin ruptured	18.92°
S3	0.1517	4171.58	212.46	z-pin ruptured	25.86°
S4	0.1978	4006.65	204.06	z-pin ruptured	13.04°
Average	\	3796.35	193.35	\	\

**Table 2:** Material properties of C/SiC composite.

**Moduli** **(GPa)**	***E*_11_ = *E*_22_**	***E*_33_**	***ν*_12_**	***ν*_13_ = *ν*_23_**	***G*_12_**	***G*_13_ = *G*_23_**
	92.14	40.00	0.01	0.01	23.18	23.18
**Strength** **(MPa)**	***X_T_* = *Y_T_***	***X_C_* = *Y_C_***	***Z_T_***	***Z_C_***	***S*_12_**	***S*_13_ = *S*_23_**
	238.91	409.40	60.31	120.19	114.53	130.00

**Table 3:** Material degradation method used in the model.

Failure Patterns	Warp Fiber Bundle	Weft Fiber Bundle	Interlaminar Matrix
Tensile failure	$\left[E_{11},G_{12},G_{13},\nu_{12},\nu_{13}\right]\times 0.01$	$\left[E_{22},G_{12},G_{23},\nu_{12},\nu_{23}\right]\times 0.01$	$\left[E_{33},G_{13},G_{23},\nu_{13},\nu_{23}\right]\times 0.01$
Compressive failure	$\left[G_{12},G_{13}\right]\times 0.1$	$\left[G_{12},G_{23}\right]\times 0.1$	$\left[G_{13},G_{23}\right]\times 0.1$

**Table 4:** Cohesive parameters used to model SiC deposition in DZC.

$K_{nn}$ (MPa/mm)	$K_{ss}$ (MPa/mm)	$\sigma_{max}$ (MPa)	$\tau_{max}$ (MPa)	$G_{Ic}$ (mJ/mm^2^)	$G_{IIc}$ (mJ/mm^2^)	Viscosity Coefficient
679,800	283,240	9.5	8.8	0.1	0.1	0.001

## Data Availability

Data is contained within the article.
